# Internalization of nanopolymeric tracers does not alter characteristics of placental cells

**DOI:** 10.1111/jcmm.12820

**Published:** 2016-03-14

**Authors:** Paolo Bigini, Elisa R. Zanier, Silvia Saragozza, Simona Maciotta, Pietro Romele, Patrizia Bonassi Signoroni, Antonietta Silini, Francesca Pischiutta, Eliana Sammali, Claudia Balducci, Martina B. Violatto, Laura Talamini, David Garry, Davide Moscatelli, Raffaele Ferrari, Mario Salmona, Maria Grazia De Simoni, Federico Maggi, Giuseppe Simoni, Francesca Romana Grati, Ornella Parolini

**Affiliations:** ^1^ IRCCS‐Istituto di Ricerche Farmacologiche “Mario Negri”MilanoItaly; ^2^ R&D UnitTOMA Advanced Biomedical Assays S.p.A.Busto ArsizioVareseItaly; ^3^ Centro di Ricerca “E. Menni”Fondazione Poliambulanza Istituto OspedalieroBresciaItaly; ^4^ Centre for BioNano InteractionsSchool of Chemistry and Chemical BiologyUniversity College DublinBelfieldDublinIreland; ^5^ Department of Chemistry, Material and Chemical Engineering“Giulio Natta” Politecnico di MilanoMilanItaly; ^6^ Institute for Chemical and BioengineeringETH ZurichZurichSwitzerland; ^7^ Fondazione IRCCS‐Istituto Neurologico Carlo BestaMilanItaly

**Keywords:** cell tracing, nanoparticles, mesenchymal stem/stromal cells, placenta, amnion, chorion, hypoxia, ischaemia

## Abstract

In the cell therapy scenario, efficient tracing of transplanted cells is essential for investigating cell migration and interactions with host tissues. This is fundamental to provide mechanistic insights which altogether allow for the understanding of the translational potential of placental cell therapy in the clinical setting. Mesenchymal stem/stromal cells (MSC) from human placenta are increasingly being investigated for their potential in treating patients with a variety of diseases. In this study, we investigated the feasibility of using poly (methyl methacrylate) nanoparticles (PMMA‐NPs) to trace placental MSC, namely those from the amniotic membrane (hAMSC) and early chorionic villi (hCV‐MSC). We report that PMMP‐NPs are efficiently internalized and retained in both populations, and do not alter cell morphofunctional parameters. We observed that PMMP‐NP incorporation does not alter *in vitro* immune modulatory capability of placental MSC, a characteristic central to their reparative/therapeutic effects *in vitro*. We also show that *in vitro*, PMMP‐NP uptake is not affected by hypoxia. Interestingly, after *in vivo* brain ischaemia and reperfusion injury achieved by transient middle cerebral artery occlusion (tMCAo) in mice, iv hAMSC treatment resulted in significant improvement in cognitive function compared to PBS‐treated tMCAo mice. Our study provides evidence that tracing placental MSC with PMMP‐NPs does not alter their *in vitro* and *in vivo* functions. These observations are grounds for the use of PMMP‐NPs as tools to investigate the therapeutic mechanisms of hAMSC and hCV‐MSC in preclinical models of inflammatory‐driven diseases.

## Introduction

Tracking stem cells in a three‐dimensional environment is essential for a comprehensive analysis of tissue regeneration. Nanoscale materials such as gold, iron oxides and fluorochromes alone or with other carriers (*e.g*. polymeric nanoparticles and liposomes), have been applied to detect or label cells and tissues of interest both *in vitro* and *in vivo* 1, 2, 3, 4. Polymers are attracting a growing interest as a result of their great plasticity and the possibility to fine‐tune their physico‐chemical properties, their degree of functionalization and their biocompatibility.

Ideally, stem cells for regenerative medicine should be found in abundant quantities, harvested by non‐invasive procedures and then transplanted safely and effectively to autologous or allogeneic hosts. Mesenchymal stem/stromal cells (MSC) can be isolated from almost any tissue but, over the past decade, those derived from the placenta have sparked interest among researchers and clinicians for their unique characteristics 5. The human term placenta offers several advantages over other conventional MSC sources (*i.e*. bone marrow, adipose tissue), such as its ease and non‐invasive procurement, its abundance and its uncritical ethical status. *In vitro* and *in vivo* studies have proven the therapeutic potential of placenta‐derived cells and, in particular, of MSC isolated from different regions of human placenta such as the amniotic membrane (hAMSC) and the chorionic villi (hCV‐MSC). Their differentiation capability towards osteogenic, chondrogenic and adipogenic lineages has been demonstrated *in vitro* 6, 7, 8. However, the most well‐studied characteristics at the basis of their therapeutic potential are their immune modulatory properties. We have previously demonstrated that both hAMSC and conditioned medium (CM‐hAMSC) derived from the culture of these cells are able to reduce T‐cell proliferation induced by alloantigens, T‐cell receptor cross‐linking or mitogens 9, 10, and induce T‐cell polarization with a reduction in Th1 response and an increase in T‐regulatory cells 11. Furthermore, the monocyte‐macrophage compartment is also influenced by hAMSC. Specifically, we have recently demonstrated that hAMSC block monocyte differentiation towards dendritic cells and induce their macrophage differentiation 12. MSC isolated from placental villi exert similar immune modulatory effects both *versus* T cells 13 and monocytes 14. The immune modulatory potential of placenta‐derived MSC has been considered to be at the basis of the paracrine mechanisms responsible for the therapeutic effect in inflammatory‐based disorders, such as fibrosis 15, 16 and autoimmune diseases 17. Mounting data indicate brain and systemic immune activation as central components of brain injury progression after stroke. Thus, considering their immunomodulatory properties, MSC are becoming important therapeutic candidates for acute brain injury.

An issue which needs to be addressed to monitor placental MSC *in vivo* is the potential repercussion of tracers on these cells. Thus, herein we sought to analyse the effects of poly (methyl methacrylate) nanoparticles (PMMA‐NPs) on hAMSC and hCV‐MSC. First, we evaluated if nanopolymeric tracers could be efficiently internalized and retained in both MSC populations, and if they altered the morphofunctional parameters and immune modulatory capability of MSC. Second, we investigated if the nanoparticles affected the MSC response to hypoxia *in vitro* and if MSC retain therapeutic potential after brain ischaemia *in vivo*.

## Materials and methods

### Ethics statements

All samples used in this study were obtained after informed consent from donors. For human term placenta and peripheral blood samples, written informed consent to collect placenta and isolate human amniotic‐derived cells was obtained from each single donor according to the guidelines of Ethical Committee of the Catholic Hospital (CEIOC). Chorionic villi (CV) samples from prenatal diagnostic villocentesis, obtained at the first trimester of gestation, were used after maternal consent. The study was reviewed and approved by the TOMA Laboratory Institutional Review Board (Institutional Review Board project No. 0000009; December 22, 2011).

### Human MSC isolation, growth and cryopreservation

Human term placentas (*n* = 15) were processed within approximately 8 hrs after birth. The amnion was manually separated from the chorion and washed extensively in saline sodium chloride 0.9% with 100 U/ml penicillin and 100 μg/ml streptomycin (herein referred to as P/S, both from Sigma‐Aldrich, St. Louis, MO, USA) and 2.5 mg/ml amphotericin B (Sigma‐Aldrich, USA). Afterwards, the amnion was cut into small fragments (~3 × 3 cm) which were decontaminated by a brief incubation in saline sodium chloride 0.9% + 2.5% Betadine – 0.25% Iodopovidone (Betadine‐Esojod 10%, Ecolab‐Esoform, Italy), and 3 min. in PBS (Sigma‐Aldrich, USA) containing 500 U/ml penicillin, 500 μg/ml streptomycin, 12.5 μg/ml amphotericin B, and 1.87 mg/ml Cefamezin (Teva, Italy). The amnion fragments were then incubated for 9 min. at 37°C in HBSS (Sigma‐Aldrich, USA) containing 2.5 U/ml dispase (VWR, Milan, Italy). After a 3–5‐min. resting period at room temperature in RPMI 1640 medium (Sigma‐Aldrich, USA) supplemented with 10% heat‐inactivated foetal bovine serum (FBS; Sigma‐Aldrich, USA), 2 mM L‐glutamine (Sigma‐Aldrich, USA) and P/S, the fragments were digested with 0.94 mg/ml collagenase (Roche, Mannheim, Germany) and 0.01 mg/ml DNase (Roche) for 2.5 hrs at 37°C. Amnion fragments were removed and mobilized cells were passed through a 100 μm strainer (BD Falcon, Bedford, MA, USA), and collected by centrifugation at 300 × g for 10 min. The cell suspension was filtered a second time with a 70‐μm strainer (BD Falcon). Cells were frozen in 90% heat‐inactivated foetal bovine serum and 10% DMSO (Sigma‐Aldrich, USA) in liquid nitrogen until use.

Human CV (*n* = 15) were frozen at the moment of selection in a cGMP solution containing 5% bovine serum albumin (BSA; Sigma‐Aldrich, Milan, Italy), 10% DMSO (Sigma‐Aldrich, Italy) and physiological solution. At the moment of hCV‐MSC isolation, chorionic villi were rapidly thawed and digested for 10 min. at room temperature with 0.125 g/l pronase E (Merck KgaA, Darmstadt, Germany). The samples were resuspended in HBSS medium (Lonza, Basel, CH, Switzerland) and centrifuged at 500 × g for 10 min. Subsequently, the pellet was treated with 0.1 g/l collagenase (Sigma‐Aldrich, Italy) for 45 min. at 37°C, resuspended in HBSS medium and centrifuged as described above. The pellet obtained was resuspended in CHANG medium C (Irvine Scientific T101‐059, Santa Ana, CA, USA) supplemented with 2 mM L‐glutamine (Biowest LLC, Kansas City, MO, USA), P/S (Lonza), and 100× Fungizone^®^ solution (Gibco, Milan, Italy).

To obtain hAMSC or hCV‐MSC at different passages, freshly isolated or thawed cells were plated in flasks (Corning, NY, USA) at a density of 10,000 cells/cm^2^ in CHANG Medium C supplemented with 2 mM L‐glutamine and P/S. Upon reaching subconfluency, adherent cells were washed in PBS, detached with 0.25% trypsin (Sigma‐Aldrich, Italy) and then subcultured at a density of 1 × 10^5^ cells/cm^2^.

### Nanoparticle synthesis and characterization

Poly (methyl methacrylate) nanoparticles (from now on referred to as NPs) were obtained from a co‐polymerization between methyl methacrylate (MMA) and a macromonomer of 2‐hydroxyethyl methacrylate covalently bound to Rhodamine b (RhB), through an emulsion‐freeradical polymerization process, as previously described 18. For all experiments, MSC were incubated with 200 nm RhB‐positive NPs (number of NPs/ml H_2_O = 1.53 × 10^13^; polymer concentration = 50 mg/ml). Details on synthesis and NP characterization have been widely reported in our previous study 19.

### Cellular internalization and cytotoxicity

The hAMSC and hCV‐MSC were seeded on round glass slides in 24‐well plates at the concentration of 20,000 cells/ml and left to adhere for 24 hrs. Longitudinal analysis of NP internalization was carried out incubating two different concentrations of NPs (1.25 × 10^10^ NPs/ml and 2.5 × 10^10^ NPs/ml) for 6, 24 and 96 hrs. In addition, to confirm that NPs remained in cells after incubation, a wash‐out experiment was carried out by incubating NPs for 96 hrs, then removing the medium with NPs and replacing it with fresh medium without NPs for 72 hrs. For all experiments, at the end of the incubation, cell nuclei were stained with Hoechst‐33258 (2 mg/ml in PBS) for 40 min. and then fixed with 4% paraformaldehyde in PBS as previously described 20. Three replicates were used for each concentration and time of incubation; additional slides without NPs were added as controls. Ten fields of view for each experimental condition (40× magnification) were captured by a Fluoview microscope BX61 (Olympus, Tokyo, Japan) with confocal FV500 system equipped with specific lasers λexc = 405 nm for Hoechst‐33258 and λexc = 546 nm to visualize the specific signal associated with NPs‐RhB. The quantification of NPs was expressed as the average of RhB‐related signal (red pixels) for each single cell. Nuclei counting and quantification of RhB were carried out by a dedicated cell segmentation software (TissueQuest, TissueGnosticGmbH, Wien, Austria) as previously described 21. To assess the internalization of NPs in the cell cytoplasm, z‐stack acquisition (one focal plane acquisition every 0.2 μm on the *z*‐axis, 30 serial planes for each cell) and three‐dimension image reconstruction were performed by Imaris 5.0 (Bitplane AG, Zurich, Switzerland) software. To evaluate a possible cytotoxic effect of 200 nm NPs, cell viability and growth curve assays were performed by seeding hAMSC and hCV‐MSC in the wells with NPs, as described above. Cells were harvested at each time‐point and the viability and growth rate were evaluated by automatic cell counter Vi‐Cell XR (Cell Viability Analyzer, Beckman Coulter, Germany), as previously described 22.

### High‐content screening

Briefly, 5 × 10^3^ hAMSC and hCV‐MSC were seeded per well in 96‐well plates. Twenty‐four hours later, cells were incubated with 1.25 × 10^10^ and 2.5 × 10^10^ NPs/ml respectively, for 96 hrs. Untreated cells were used as controls. At the end of incubation, the medium was removed and replaced with medium containing the following dyes: Hoechst‐33342 (400 nM), Lysotracker green (200 nM) and TOPRO‐3 (800 nM). One hour after this second incubation, cells were analysed by High Content Analysis using the Arrayscan VTI 740 (Thermo Fisher Scientific, Pittsburgh, USA) as previously reported by our group 23.

### Flow cytometry

To evaluate cell‐surface marker expression, hAMSC and hCV‐MSC were washed with FACS buffer (0.1% sodium azide, Sigma‐Aldrich, Italy; and 0.1% BSA, Sigma‐Aldrich, Italy in PBS). The following antibodies specific for human markers associated with mesenchymal and haematopoietic lineages used were: CD73 (clone AD2), CD44 (clone L178), CD90 (clone 5E10), CD13 (clone L138), CD45 (clone 2D1) (all from BD Biosciences, San Jose, CA, USA) and CD105 (clone SN6; Serotec, Oxford, UK). Dead cells were gated out by propidium iodide staining (for cell‐surface staining). The samples were analysed on a FACS Calibur cytometer, and the data were processed using CellQuest software (BD Biosciences).

### MSC differentiation

Twenty‐four hours after NP loading, hAMSC and hCV‐MSC were counted and differentiated toward chondrogenic, adipogenic and osteogenic lineages. For chondrogenic differentiation, 2.5 × 10^5^ cells were grown for 2–3 weeks in tubes with hMSC Chondrogenic bulletkit (Lonza) supplemented with 10 ng/ml TGFβ3 (Lonza). The differentiation was detected with Alcian Blue staining (Sigma‐Aldrich, Italy). For adipogenic differentiation, cells were plated at 5 × 10^3^/cm^2^ and when 80% confluency was reached, differentiation was induced using hMSC Adipogenic bulletkit (Lonza). Three weeks after the start of differentiation, the cytoplasmatic lipid vacuoles were stained with Oil red solution (Sigma‐Aldrich, Italy). For osteogenic differentiation, cells were plated at 5 × 10^3^/cm^2^ and when 80% confluency was reached, differentiation was induced using hMSC Osteogenic bulletkit (Lonza). Calcium deposition was detected 3 weeks after the start of differentiation by Alizarin red staining (Sigma‐Aldrich, Italy). CHANG medium C by itself and cells without NPs were used as controls.

### Immunomodulation

Peripheral blood mononuclear cells (PBMC) were obtained from heparinized whole blood samples or buffy coats from healthy individuals using density gradient centrifugation (Lymphoprep; Axis‐Shield, Oslo, Norway). Immune modulation was evaluated as the ability of hAMSC and hCV‐MSC, either with or without NPs, to inhibit the proliferation of PBMC activated with anti‐CD3, as previously described 9. Peripheral blood mononuclear cell (1 × 10^5^/well in 96‐well‐plate) were stimulated with 12.5 ng/ml (final concentration) anti‐CD3 (Orthoclone OKT3, Janssen‐Cilag, Milan, Italy). All conditions were performed in triplicate in RPMI 1640 medium (Cambrex, Verviers, Belgium) supplemented with 10% heat‐inactivated FBS, 2 mM L‐glutamine, and P/S. After 3 days of culture, lymphocyte proliferation was assessed by adding 0.67 μCi of [3H]‐thymidine (Perkin Elmer, Milan, Italy) per well. Sixteen to 18 hrs after the addition of thymidine, cell cultures were harvested with a Filtermate Harvester (Perkin Elmer, Milan, Italy), and thymidine incorporation was measured using a microplate scintillation and luminescence counter (Top Count NXT; PerkinElmer). The following concentrations of passage 4 (P4) hAMSC and hCV‐MSC (with or without NPs) were used: 1 × 10^5^, 0.5 × 10^5^, 0.25 × 10^5^, 0.125 × 10^5^, 0.0625 × 10^5^ cells/well. Placental cells were seeded in 96‐well plates in contact with 1 × 10^5^ PBMC. The hAMSC, hCV‐MSC and PBMC alone were used as controls to assess basal proliferation.

### *In vitro* hypoxic challenge

The P3 hAMSC and hCV‐MSC, each from five different donors, were seeded in CHANG medium C at 37°C in 5% CO_2_ atmosphere. After 3 days, hAMSC and hCV‐MSC were incubated with or without NPs (2.5 × 10^10^ NPs/ml) for 1 day. Cells were seeded in 24‐well plates at the concentration of 5 × 10^3^ cells/cm^2^ and left to adhere for one additional day. Cells were then incubated in a hypoxic chamber for 3 days (InvivO_2_ 400, Baker Ruskinn, Bridgend, UK) at the following gas concentrations: 1% O_2_, 5% CO_2_ and 94% N_2_. Control cells were maintained in a normoxic incubator for 3 days. For each cell line, viable cell number (Trypan blue dye exclusion method) was quantified after 3 days, and normalized over its proper control (representing 100% value). The values of the five donors from each source were averaged.

### Animal model

Male C57BL/6J mice (9 weeks of age; Harlan Laboratories, Udine, Italy) were housed in a specific pathogen‐free vivarium at a constant temperature (21 ± 1°C) with a 12 hrs light–dark cycle and *ad libitum* access to food and water. Procedures involving animals and their care were conducted in conformity with institutional guidelines that are in compliance with national and international laws and policies (Italian Governing Law:D.lgs 26/2014; Authorisation n.19/2008‐A issued March 6, 2008 by Ministry of Health; Mario Negri Institutional Regulations and Policies providing internal authorisation for persons conducting animal experiments: Quality Management System Certificate – UNI EN ISO 9001:2008 – Reg. No. 6121; the NIH Guide for the Care and Use of Laboratory Animals (2011 edition) and EU directives and guidelines (EEC Council Directive 2010/63/UE). The Statement of Compliance (Assurance) with the Public Health Service Policy on Human Care and Use of Laboratory Animals have been recently reviewed (9/9/2014) and will expire on September 30, 2019 (Animal Welfare Assurance #A5023‐01).

This specific protocol followed the ARRIVE guidelines and was approved by the IRCCS‐IRFMN Animal Care and Use Committee and by the Italian ‘Istituto Superiore di Sanità’ (code: 32/12D).

Mice were randomly allocated for surgery and treatments by a list randomizer (www.random.org/list), taking care to distribute them equally across experimental days. All surgeries were performed by the same investigator. All behavioural evaluations were performed by investigators unaware of injury/treatment status of the animals.

### Transient middle cerebral artery occlusion

Anaesthesia was induced by 3% isoflurane inhalation, and maintained by 1–1.5% isoflurane inhalation, in an N_2_O/O_2_ (70/30%) mixture. Transient ischemia was achieved by middle cerebral artery occlusion (tMCAo) using a siliconized filament (7‐0; Doccol Corporation, Sharon, MA, USA) introduced into the internal carotid artery and advanced to block the MCA 24 for 60 min. Sham‐operated mice received identical anaesthesia and surgery without artery occlusion.

### hAMSC administration

Human AMSC were resuspended in PBS and cell concentration was adjusted to 1 × 10^6^ hAMSC in 200 μl of PBS containing 1 μg of heparin. Twenty‐four hours after surgery, 1 × 10^6^ hAMSC or PBS alone were administered intravenously through the tail vein. To evaluate MSC distribution after intravenous delivery, hAMSC were labelled with NPs/Hoechst‐33258 as previously described 18. Human AMSC were intravenously infused in sham/tMCAo mice 24 hrs after surgery. Mice (*n* = 2 group/time point) were sacrificed immediately, 2 or 24 hrs after infusion.

To evaluate MSC efficacy after ischemic injury, hAMSC were intravenously infused in sham/tMCAo mice 24 hrs after surgery. Mice (*n* = 8 group) were tested for cognitive deficits (by novel object recognition) after 5 weeks.

### Assessment of functional outcome

Cognitive deficits were evaluated 5 weeks after tMCAo by novel object recognition (NOR) test that assesses long‐term recognition memory. In the NOR test, mice are introduced into a grey Perspex square arena surrounded by walls (40 × 40 × 30 cm) with the floor divided into 25 squares (8 × 8 cm), placed in a specific room separated from the operator's room. The task started with a habituation trial during which the animals were placed in the empty arena for 5 min., and their movements were recorded as the number of line crossings, which provide an indication of motor activity. Mice were tested following a predefined scheme so to precisely maintain the 24 hrs of retest for each mouse. The next day, mice were again placed in the same arena containing two identical objects (familiarization phase). Exploration was recorded in a 10 min. trial by an investigator blinded to surgery and to treatment. Sniffing, touching and stretching the head towards the object at a distance of no more than 2 cm were scored as object investigation. Twenty‐four hours later (test phase), mice were again placed in the arena containing two objects, one of the objects presented during the familiarization phase (familiar object) and a new different one (novel object), and the time spent exploring the two objects was recorded for 10 min. The following objects were used: a black plastic cylinder (4 × 5 cm), a glass vial with a white cup (3 × 6 cm) and a metal cube (3 × 5 cm). Results were expressed as discrimination index (DI), that is, (seconds spent on novel ‐ seconds spent on familiar)/(total time spent on objects). Animals with no memory impairment spent a longer time investigating the novel object, giving a higher DI 25.

### Transcardial perfusion

At indicated time‐points, mice were deeply anaesthetized with Equitensin (120 μl/mouse i.p.) and transcardially perfused with 20 ml of PBS, 0.1 mol/l, pH 7.4, followed by 50 ml of chilled paraformaldehyde (4%) in PBS. The brains, lungs, spleen and liver were collected and transferred to 30% sucrose in PBS at 4°C overnight for cryoprotection. The organs were then rapidly frozen by immersion in isopentane at −45°C for 3 min., sealed in vials, and stored at −70°C until use.

### Statistical analysis

All data are expressed as mean ± S.D. All statistical analyses were performed with GraphPad Prism version 6.00 for Windows (Graph‐Pad Software, San Diego, CA, USA). Comparison of counts per minute (cpm) values between hAMSC or hCV‐MSC and hAMSC or hCV‐MSC + NPs and PBMC activated with anti‐CD3 was performed with paired *T*‐test; analysis between hAMSC or hCV‐MSC and hAMSC or hCV‐MSC + NPs was performed with unpaired *T*‐test. Memory performance was analysed by a one‐way anova.

## Results

### NP internalization and viability of hAMSC and hCV‐MSC after internalization

**Figure 1:**
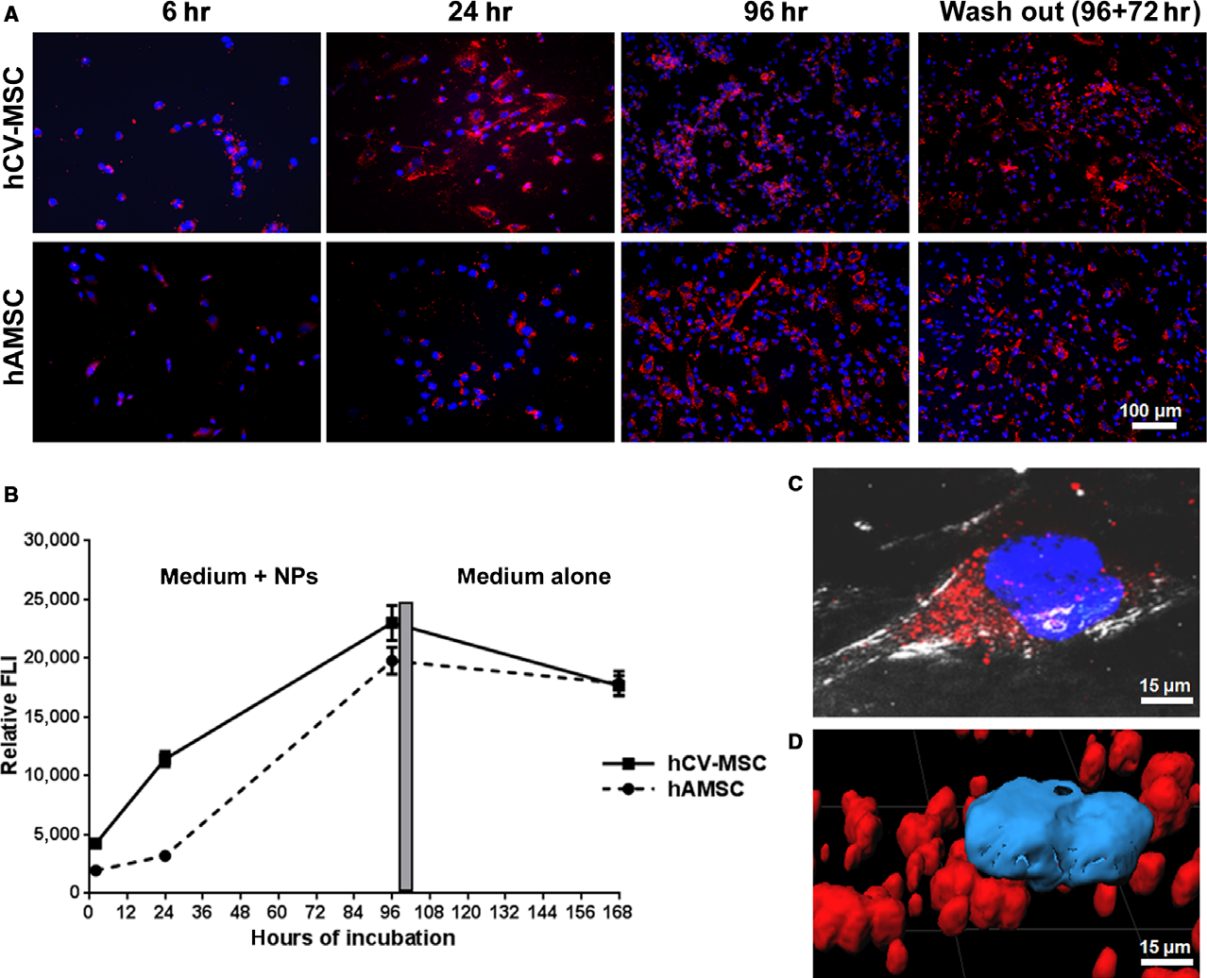
NP internalization. (**A**) Representative microscopy images showing internalization of NPs (red signal) in hAMSC (upper panels) and hCV‐MSC (lower panels) after 6, 24, 96 hrs of incubation and after 72 hrs of wash out. The blue signal is related to the staining of nuclei with the Hoechst‐33258; the red staining is associated with RhB. (**B**) Diagrams showing the trend of staining intensity associated with RhB in both MSC populations at different time‐points. Each point represents the average of the percentage of fluorescent surface normalized to the total number of Hoechst‐33258 positive cells. (**C**) A single cell image confirms the internalization of NPs in perinuclear region inside the cell. (**D**) A 3D reconstruction of treated cells, reported at high magnification, confirms association between NPs and Hoechst‐33258.

Internalization experiments confirmed the ability of NPs to be internalized by both hAMSC and hCV‐MSC in a fast and efficient way. Furthermore, a progressive time‐dependent accumulation was observed for both cell types (Fig. 1A). Very interestingly, the 72‐hr wash‐out did not markedly modify the overall signal associated with RhB. The permanence in cells was somehow expected and is likely because of a segregation of NPs in endosomal vesicles that almost completely limited their leakage 19. The quantification of the signal at different time‐points and in the two different cell types confirmed the observational evidence (Fig. 1B). A significant time‐dependent increase in signal per cell was observed for each group. On the contrary, in the two cell types after wash‐out, neither the trend of signal increase, nor the RhB‐related fluorescent signal, were found statistically different. The overlap between interferential contrast (Nomarski) and fluorescence signal at a higher magnification confirmed that the signal associated with NPs is confined as spots in the cytoplasm, near the perinuclear region (Fig. 1C). This further strengthens the hypothesis of a vesicle‐mediated internalization into the cells. To confirm that the fluorescent signal actually penetrated into the cytoplasm, a 3D reconstruction was carried out (Fig. 1D).

**Figure 2:**
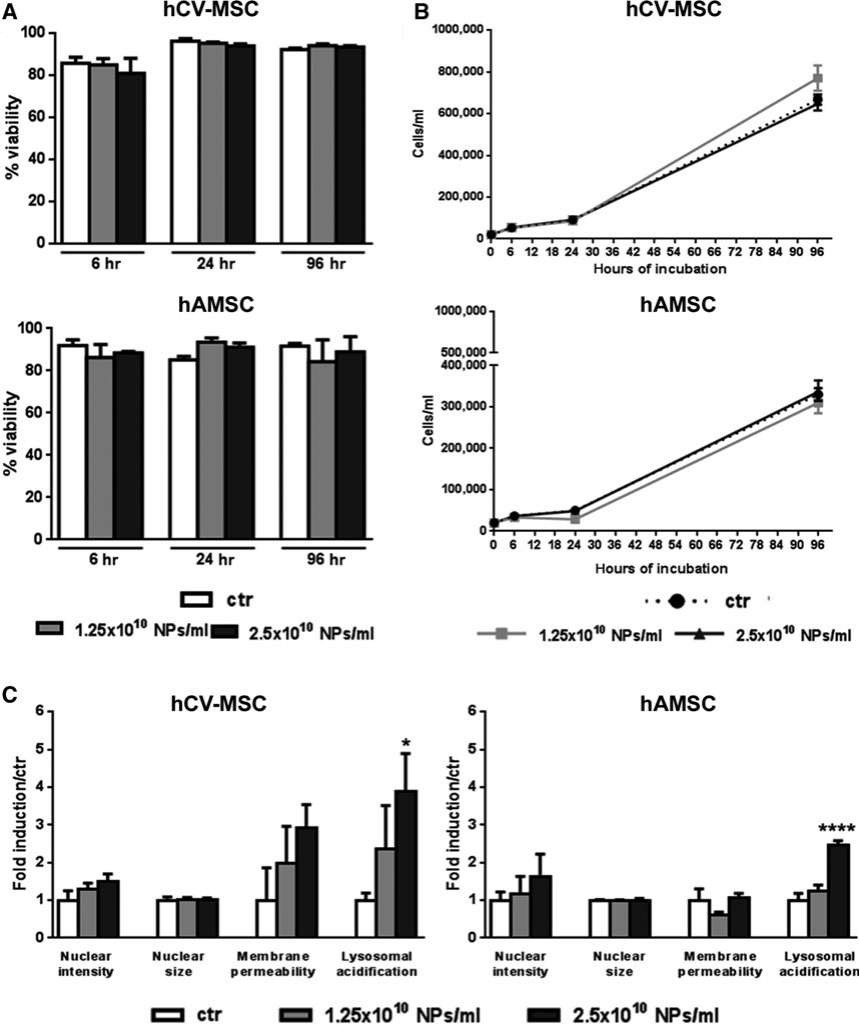
MSC viability and growth rate. Viability graphs (**A**) and growth curves (**B**) represent both MSC populations exposed to different concentrations of NPs at different time‐points. (**C**) High content analysis performed in hAMSC (right histogram) and hCV‐MSC (left histogram) exposed for 72 hrs to vehicle (ctr) or increasing concentrations of NPs. This approach allowed to assess changes in nuclear morphology (Hoechst 33342), plasma membrane integrity (TOPRO‐3) and lysosomal acidification (Lysotracker green). Signal values were normalized to those measured in control cells (white bars). All data are represented as mean ± S.D., statistical analysis was performed by a one‐way anova. *P < 0.05; ****P < 0.0001.

Once established that NPs were able to enter cells, their interaction with host cells, in terms of survival and growth, was investigated. As shown in Figure 2A, no relevant difference of hAMSC (top) and hCV‐MSC (bottom) viability was observed among the two concentrations used in this study (1.25 × 10^10^ NPs/ml and 2.5 × 10^10^ NPs/ml). Furthermore, the proliferation of both MSC populations was not inhibited by NPs, as shown by a consistent increase in cell number during NP incubation (Fig. 2B). Taken together, these results confirm the tracking efficiency, safety and biocompatibility of NPs in both hAMSC and hCV‐MSC.

To evaluate if NP internalization could lead to more subtle alterations of the cell homoeostasis, High Content Analysis was performed in both hAMSC and hCV‐MSC incubated for the longest experimental time‐point at the two concentrations used for the internalization experiments (Fig. 2C).

Our data confirmed a mild effect produced by NP internalization in both cell types. At both concentrations tested, NPs did not cause alterations in any of the two important nuclear parameters (size and chromatin intensity). A small, but insignificant increase in membrane permeability was found in hCV‐MSC exclusively in a concentration‐dependent manner (Fig. 2C). On the other hand, the highest dose led to a significant increase in lysosomial acidification in both cell lines (Fig. 2C). Based on the results obtained, all subsequent experiments were performed with the lowest dose of NPs.

### Effect of NP internalization on MSC morphofunctional parameters

**Figure 3:**
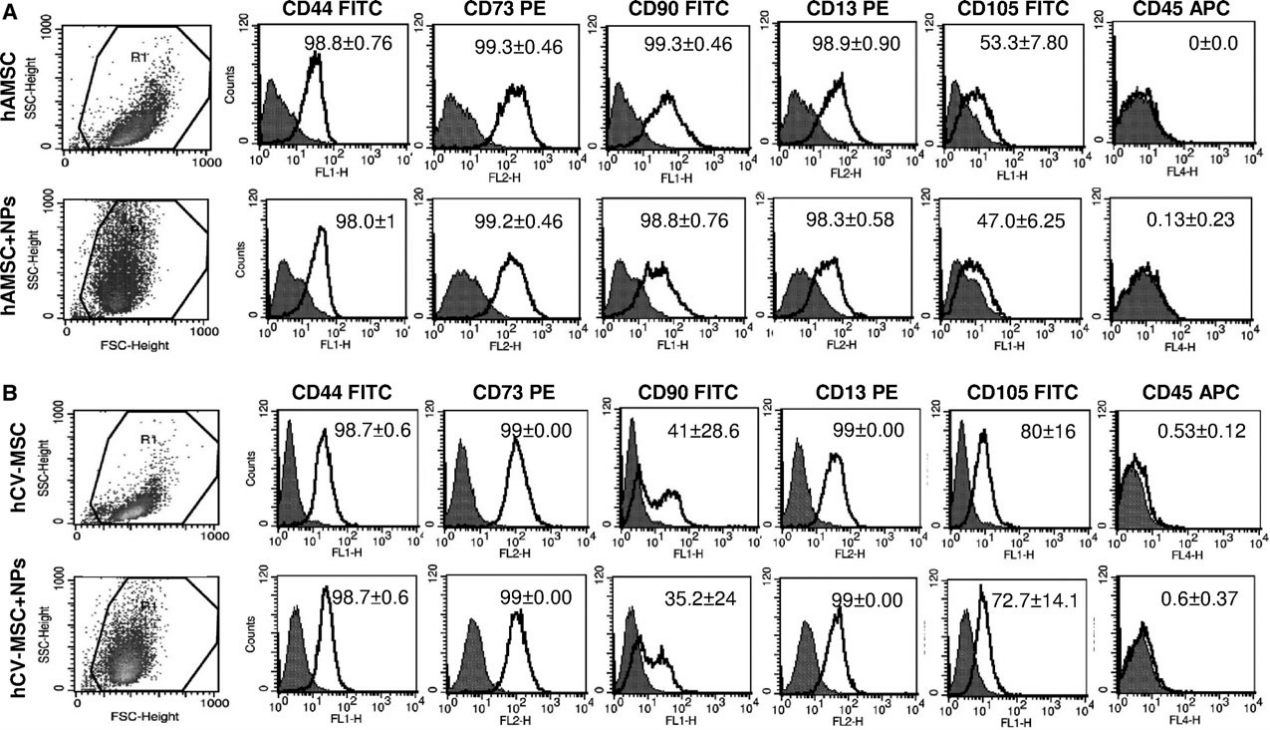
Phenotype of hAMSC and hCV‐MSC in the presence of NPs. Physical parameters and phenotype analysis of hAMSC (**A**) and hCV‐MSC (**B**) were analysed by flow cytometry before and after NP incorporation. Physical parameters were determined by forward‐scatter (FSC) and side‐scatter (SSC) properties. Phenotype analysis with corresponding monoclonal antibodies (white histograms) or isotype‐matched IgG controls (grey histograms) are shown. The histograms show one representative experiment (*n* = 3), and the mean percentage of positive cells with standard deviation is indicated in each plot.

The International Society of Cellular Therapy previously defined the general minimal criteria for MSC 26. Subsequently, the consensus from the First International Workshop on Placenta‐Derived Stem Cells established the nomenclature and characteristics for the identification of MSC from the foetal membranes of placenta 5. These comprise the differentiation potential toward one or more lineages, including osteogenic, adipogenic or chondrogenic lineages, and specific surface antigen expression, such as the positive expression of CD73, CD90 and CD105, and low or absent expression of hematopoietic markers and HLA‐DR 5. Thus, we sought to investigate whether NPs affected these properties. As shown in Figure 3, analysis of the physical parameters, such as size, granularity and complexity, on the basis of their forward and side light scatter characteristics showed that the size/complexity of hAMSC and hCV‐MSC is altered after the incorporation of NPs, as expected.

The analysis of hAMSC phenotype at passage 4 (Fig. 3A) confirmed the general mesenchymal features, as shown by high expression of CD44 (mean ± S.D.: 98.8 ± 0.76), CD73 (99.3 ± 0.46), CD90 (99.3 ± 0.46), CD13 (98.9 ± 0.9), CD105 (53.3 ± 7.8) and lack of CD45 expression. The incorporation of NPs showed no significant alterations in the phenotype of hAMSC [CD44 (98.0 ± 1), CD73 (99.2 ± 0.46), CD90 (98.8 ± 0.76), CD13 (98.3 ± 0.58), CD105 (47 ± 6.25), CD45 (0.13 ± 0.23)]. The analysis of hCV‐MSC phenotype at passage 4 (Fig. 3B) also confirmed their mesenchymal features, as shown by high expression of CD44 (mean ± S.D.: 98.7 ± 0.6), CD73 (99 ± 0), CD90 (41 ± 28.6), CD13 (99 ± 0), CD105 (80 ± 16) and lack of CD45 expression (0.53 ± 0.12). Once again, the incorporation of NPs showed no alterations in the phenotype [CD44 (98.7 ± 0.6), CD73 (99 ± 0), CD90 (35.2 ± 24), CD13 (99 ± 0), CD105 (72.7 ± 14.1), CD45 (0.6 ± 0.37)].

**Figure 4:**
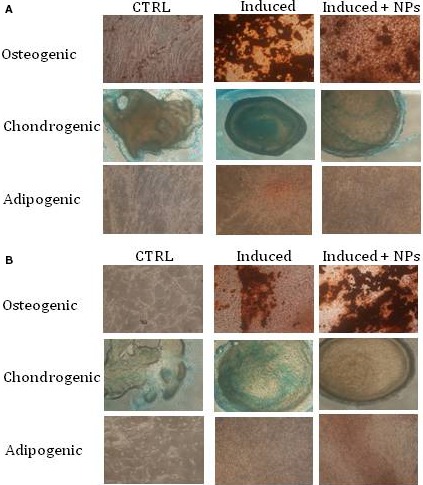
MSC differentiation after NP internalization. hAMSC (**A**) and hCV‐MSC (**B**) from three different donors were incubated with or without NPs. After 24 hrs, cell differentiation toward the osteogenic, chondrogenic, and adipogenic lineages was induced. Osteogenic differentiation was revealed with Alizarin red staining, chondrogenic differentiation with Alcian blue and adipogenic differentiation with Oil red solution.

Next, we studied if the internalization of NPs interfered with the differentiation capability of hAMSC and hCV‐MSC towards chondrogenic, adipogenic and osteogenic lineages (Fig. 4). The differentiation was induced on three different P4 cell lines for each cell type, and cells were stained 3 weeks after induction. Incorporation of NPs did not alter in any way the differentiation of hAMSC (Fig. 4A) and hCV‐MSC (Fig. 4B) towards the three lineages analysed. Altogether, these results show that NP internalization does not perturb the main morphofuntional aspects of hAMSC and hCV‐MSC.

### Effect of NP internalization on MSC immunomodulation

**Figure 5:**
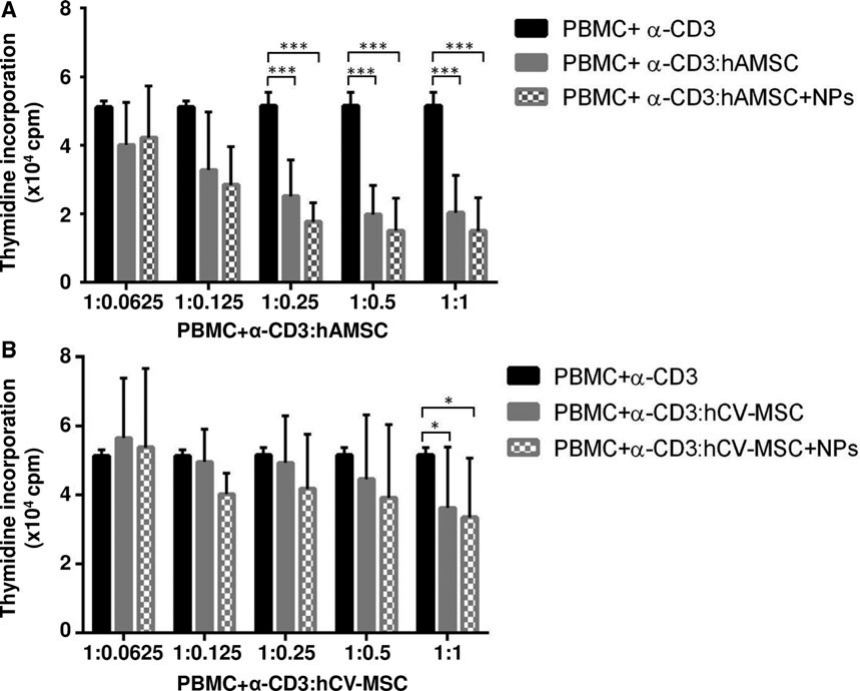
The effect of NP internalization on hAMSC and hCV‐MSC immune modulation. (**A**) Proliferation of peripheral blood mononuclear cells (PBMC) in the presence of hAMSC with or without NPs. Data are expressed as mean ± S.D. of 3 (for 1:0.0625 and 1:0.125) or 8 (for 1:0.25, 1:0.5, and 1:1) different donors. Comparison of counts per minute (cpm) values between hAMSC or hAMSC+NPs and PBMC activated with anti ‐CD3 was performed with a *T*‐test. A *P* value of less than 0.05 was considered statistically significant (***P < 0.001). (**B**) Proliferation of PBMC in presence of hCV‐MSC with or without NPs. Data are expressed as mean ± S.D. of 3 (for 1:0.0625 and 1:0.125) or 5 (for 1:0.25, 1:0.5 and 1:1) different donors. Comparison of cpm values between hCV‐MSC or hCV‐MSC+NPs and PBMC activated with anti‐CD3 was performed with a *T*‐test. A *P* value of less than 0.05 was considered statistically significant (*P < 0.05).

We have previously reported that hAMSC are able to inhibit PBMC proliferation induced by cell receptor engagement 9. Herein, we show that the incorporation of NPs does not alter the ability of hAMSC and hCV‐MSC to inhibit T‐cell proliferation (Fig. 5). T‐cell proliferation was significantly (*P* < 0.05) inhibited when anti‐CD3‐activated PBMC were cultured in contact with the three highest hAMSC concentrations tested (Fig. 5A), while hCV‐MSC showed a higher variability and were able to significantly inhibit T‐cell proliferation only at the highest concentration used (Fig. 5B). No significant differences were observed between cells with and without NPs.

### Influence of NP internalization on MSC response to hypoxia

**Figure 6:**
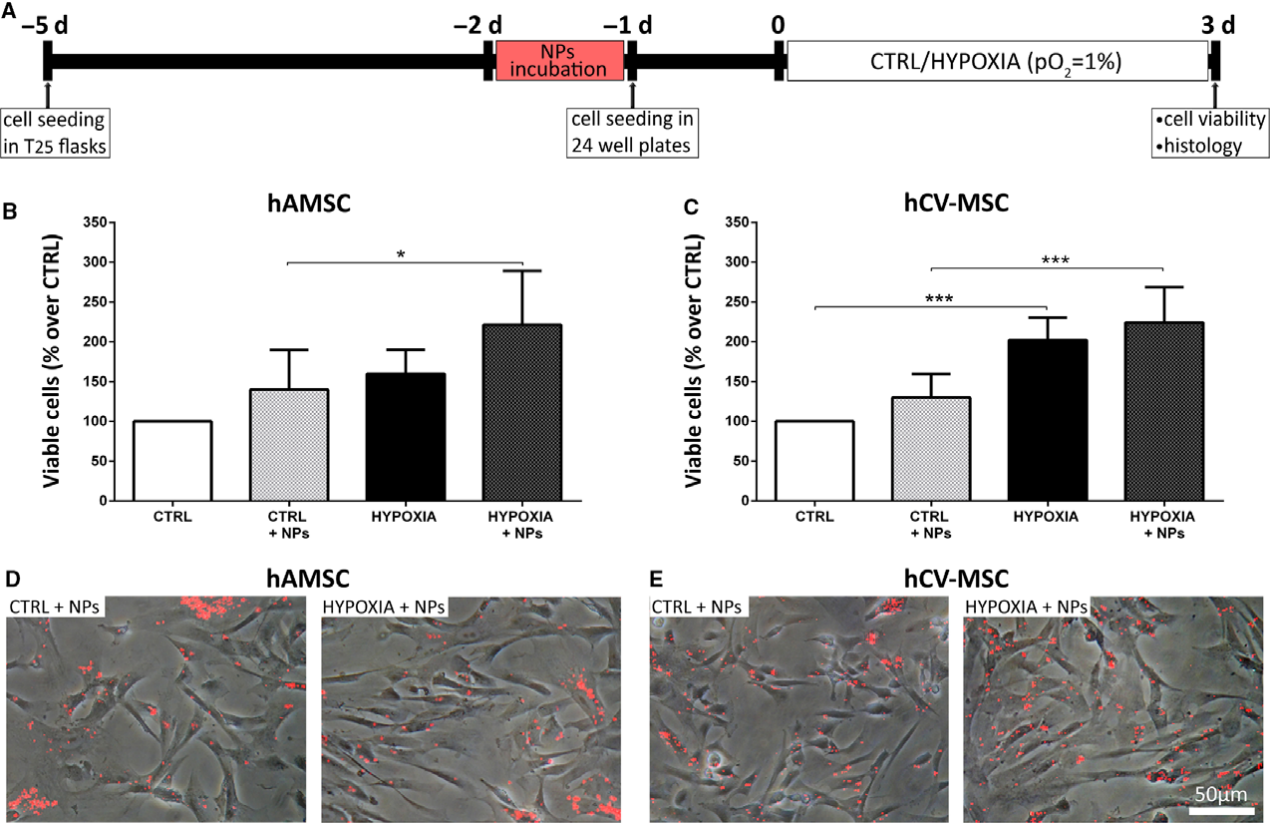
The effects of NPs on MSC response to hypoxia. (**A**) Experimental design. hAMSC and hCV‐MSC were incubated with or without NPs for 1 day and then seeded in 24‐well plates for one additional day. Cells were then incubated for 3 days under hypoxic (HYPOXIA: 95% N_2_, 5% CO_2_ and 1% O_2_) or normoxic (CTRL) conditions. Cell survival was evaluated using Trypan blue dye exclusion method. Quantification of viable cells is shown for hAMSC and hCV‐MSC in panels (**B**) and (**C**), respectively. (**D** and **E**) Internalization of NPs does not appear to be affected by hypoxia as shown in the representative microphotographs, displaying a similar cellular localization of the NPs in the different conditions. Data are reported as mean + S.D. (*n* = 5). Statistical analysis was performed by a two‐way anova followed by Tukey's post hoc test. *P < 0.05; ***P < 0.001.

To determine whether internalized NPs are able to affect the response of MSC to hypoxic conditions, hAMSC and hCV‐MSC from five different donors, with or without NPs, were incubated for 3 days in a hypoxic chamber (Fig. 6A). Compared to normoxic conditions, both hAMSC and hCV‐MSC showed an increased cell number when cultured in hypoxia (Fig. 6B and C). Nanoparticle internalization did not affect cell viability, either in control or hypoxic conditions (Fig. 6B and C). Nanoparticle uptake did not appear to be affected by hypoxia, which showed a similar cellular localization of the NPs in the different conditions (Fig. 6D and E).

### Effects of NP‐loaded hAMSC in sham or ischaemic mice

**Figure 7:**
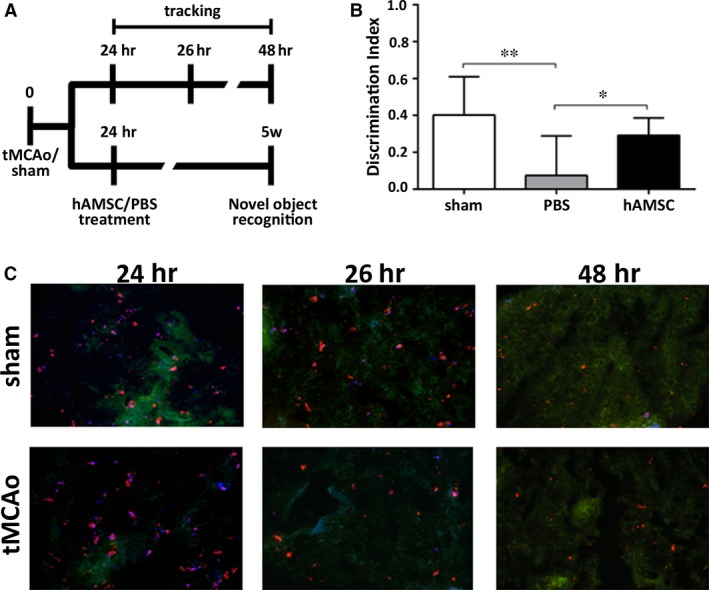
In vivo studies. (**A**) Experimental design. hAMSC or PBS were administered intravenously 24 hrs after tMCAo/sham injury. For cell tracking, tMCAo and sham mice were killed 24, 26 and 48 hrs after surgery. The efficacy study was performed by evaluating the effects of hAMSC on recovery of functions by cognitive test performed at 5 weeks after surgery. (**B**) A significant beneficial effect on long‐term recognition memory was detected in tMCAo mice treated with hAMSC compared to PBS 5 weeks after injury. Data are represented as mean ± S.D. (*n* = 12), NOR one‐way anova, followed by Sidak post‐hoc test. *P < 0.05; **P < 0.01 (**C**) Histological sections of lungs from sham or tMCAO mice treated with hAMSC. Representative microscopy images showing localization of NP‐loaded hAMSC (red signal) at different time‐points. The blue signal is related to the staining of nuclei with the Hoechst‐33258 and the red staining is associated with RhB. The green staining is related to the autofluorescence of lung tissue.

Human AMSC were selected for the preliminary investigation of their therapeutic features in tMCAo mice. To evaluate hAMSC distribution after infusion, hAMSC were labelled with NPs/Hoeschst *in vitro*, and then intravenously infused in mice 24 hrs after sham/tMCAo injury (Fig. 7A). To evaluate hAMSC efficacy after brain ischaemia, we assessed cognitive deficits by NOR test. tMCAo hAMSC mice showed a significant memory improvement compared to tMCAo PBS mice (mean ± S.D.: sham 0.40 ± 0.07; tMCAo PBS 0.07 ± 0.08; tMCAo hAMSC 0.29 ± 0.03; Fig. 7D).

As shown in Figure 7C, NP‐loaded hAMSC were detected in the lungs of sham and ischaemic mice up to 48 hrs after surgery; on the other hand, no hAMSC were found in brain, spleen or liver (data not shown).

## Discussion

Herein, we sought to investigate the feasibility of using PMMA‐NPs as tracers of placenta‐derived MSC, namely those from the amniotic membrane (hAMSC) and early chorionic villi (hCV‐MSC), focusing on the main physiological and functional features which characterize MSC 5, 26, and also their *in vivo* therapeutic potential. This is an innovative study which uses biocompatible polymeric nanoparticles as a means of tracing placental MSC. In particular we show that NPs do not substantially modify placental MSC charactersitics, such as viability, phenotype and cell differentiation. We also report that NP incorporation does not alter immunomodulatory capacity, and hAMSC loaded with NPs possess/retain therapeutic potential *in vivo*.

Similar to MSC from bone marrow 26, those from the human term placenta are characterized by minimal criteria 5, amongst these are the expression of typical mesenchymal markers and their ability to differentiate towards one or more lineages. In this study we used two different placental MSC models: one from first trimester chorionic villi readily available from villocentesis, and the second from the amniotic membrane of term placenta. We show that NPs were efficiently internalized and retained in both MSC populations, and did not alter cell viability, survival and growth. We observed an alteration in cellular complexity after NP internalization, however, this natural cellular response to a massive entry of material through endosomal uptake has been widely demonstrated in other studies which did not show any toxic effect of polymeric NPs 19, 27. These results further confirmed that cells respond to NPs. However, this perturbation does not lead to marked alterations of important subcellular mechanisms at the doses and exposure times investigated.

We observed that the typical mesenchymal cell phenotype, such as the expression of CD44, CD73, CD90, CD13, CD105 and lack of expression of the haematopoietic marker CD45, was unmodified by NPs. This is in line with others which showed that iron oxide‐PLLA particle labelling did not alter the typical surface antigen pattern of human MSC from bone marrow, as defined by ISCT, with the exception of differences in CD71 (transferrin receptor) 28.

Even though the ability of placental MSC to differentiate has been recently questioned 8, in our experimental set‐up, this property was unaltered by the incorporation of NPs. Very recently, one study reported that the incorporation of superparamagnetic iron oxide nanoparticles in ovine bone marrow‐derived MSC significantly reduced their ability to differentiate toward the chondrogenic lineage, while it had no effect on their osteogenic differentiation ability 29. Of note, a different group recently suggested toxic effects of superparamagnetic iron oxide nanoparticles as a result of the excess accumulation of iron in the ischaemic myocardium 30. On the other hand, iron oxide‐PLLA nanoparticle labelling of human bone marrow MSC was found to have no effect on their adipogenic, chondrogenic and osteogenic differentiation potential 28. Our results are in line with other groups which investigated placental MSC. For example, one study demonstrated that human umbilical cord MSC labelled with multimodal iron oxide nanoparticles with fluorescent and magnetic properties differentiated into both adipocyte‐like and osteoblast‐like cells 31. Furthermore, a very recently published study which investigated fluorescence tags on human umbilical cord MSC observed no differences in any of the three differentiation conditions between unlabeled, CM‐Dil‐labelled and GFP‐labelled cells 32.

One of the hallmark properties of placental MSC is their immune modulatory ability 9, 11, 12, 33, 34. In fact, the use of placental MSC has proven beneficial in preclinical models of diseases with underlying altered inflammatory processes 11, 17, altogether rendering these cells attractive therapeutic options. The role of MSC as modulators of immune responses is crucial for their clinical potential, making it mandatory to check that this function is preserved after labelling. Our results show that both hAMSC and hCV‐MSC retained their ability to inhibit T‐cell proliferation, even after incorporation of NPs. This is in line with a study which showed unaltered capacity of human bone marrow MSC to inhibit both T‐ and NK‐cell proliferation after NP labelling 28. In addition, conditioning MSC by a combination of interferon‐γ/tumour necrosis factor‐α was able to reinforce immunosuppressive properties, thus demonstrating that the capacity of MSC to respond to these inflammatory stimuli was unmodified after NP labelling, as revealed by their increased capacity to inhibit both T‐ and NK‐cell proliferation 28.

In the context of cell therapy, cell tracking *in vivo* is an important methodology in the development of successful therapies, and monitoring cells in preclinical studies is essential to monitor cell survival, migration, distribution and potential chimerism with host cells. Fluorescent, biocompatible and long‐lasting traceable PMMA‐NPs have been previously developed for tracking human amniotic fluid cells by *ex‐vivo* analyses 21. More recently, we have shown PMMA‐NPs are able to efficiently label umbilical cord mesenchymal stromal cells *in vitro*, and did not alter cell biodistribution or did not induce toxicity when transplanted in a mouse model of amyotrophic lateral sclerosis 18. Herein, we show that PMMA‐NPs do not alter *in vitro* properties of MSC from amnion and chorion, such as their viability, phenotype, differentiation capability and immunomodulation.

To address if NPs may differentially affect MSC in baseline or stressed conditions, we evaluated cell viability, growth and phenotype after a hypoxic stimulus (1% O_2_ for 3 days). Our observations demonstrate that hypoxia did not influence NP uploading, and that NP internalization had no adverse effect on cell viability, confirming their potential use in disease settings.

For the investigation of MSC distribution and of their therapeutic potential for the ischaemic injured brain, we focused on MSC from the amniotic membrane 35. When infused intravenously 24 hrs after sham or ischaemic‐reperfusion injury in mice, NP‐loaded hAMSC were detected in the lungs up to 48 hrs after surgery, while no cells were found in brain, spleen, or liver at that time. Even if the cells could not be detected at later time‐points in the peripheral organs, they produced long‐lasting changes as shown by the improvement in recognition memory performance 5 weeks after injury compared to tMCAo mice treated with PBS. This is in line with previous reports showing significant improvement of functional outcome following iv injection of adherent mesenchymal‐like cells isolated from human placental tissue (PDA001) 4 hrs after stroke (MCAo) in rats, when analysed 1, 3, 7 and 14 days after treatment 36. This same group reported little or no human cells in rat brains, up to 14 days after treatment 36. In a follow‐up study, they showed improved functional outcome when PDA001 was administered 24 hrs after stroke 37. Our observations provide a proof of principle for their protective effects for the ischaemic injured brain, and importantly, support the hypothesis that hAMSC act in a paracrine manner and in this model this may occur during the early‐phases of ischaemic events. This evidence prompts future studies to address the mechanisms responsible for the observed protection and to fully define if indeed hAMSC may be suitable as a therapeutic strategy for stroke.

In conclusion, PMMA‐NPs represent a suitable tool for tracking placental MSC. They are well‐tolerated and stable, and do not alter cell characteristics. PMMA‐NPs represent an important tool for preclinical and clinical studies aimed at investigating the homing and therapeutic potential of these cells. These results are promising for tracking hAMSC in different preclinical models, even during foetal development 38, 39. In addition, they are also grounds for investigating hAMSC tumour homing, considering our previous reports demonstrating the ability of hASMC to inhibit tumour cell proliferation, either directly 40 or after drug uptake and release 41.

## Conflicts of interest

The authors confirm that there are no conflicts of interest.

## Author contribution

PB and ERZ coordinated *in vitro* and *in vivo* studies and participated in drafting the manuscript; SS e SM processed hCV samples and performed differentation experiments; PR collected and processed placentas for hAMSC isolation and culture; PBS carried out flow cytometry studies and both PR and PBS participated in the collection and analysis of data; AS participated in data analysis and drafting the manuscript; FP and ES carried out *in vitro* and *in vivo* experiments and post‐mortem cell analysis; CB performed behavioural and data analysis; MBV performed NP‐cell interaction studies; LT carried out histology and confocal microscopy; DG performed High content screenings; DM and RF coordinated the synthesis and the characterization of PMMA NPs; MS and MGDS participated in project planning and in the critical reviewing of results; FM and GS participated in the study design and supervised the research; FRG participated in conceiving the study and in study design, supervised the research and managed the submission of the project to the IRB; OP participated in conceiving the study and in study design, supervised the research and participated in drafting and critically reviewing the manuscript.
